# Statement on bioinformatics and capturing the benefits of genome sequencing for society

**DOI:** 10.1186/s40246-019-0208-4

**Published:** 2019-05-29

**Authors:** Benjamin Capps, Ruth Chadwick, Yann Joly, Tamra Lysaght, Catherine Mills, John J. Mulvihill, Hub Zwart

**Affiliations:** 1 Department of Bioethics, Dalhousie University, 5849 University Avenue, CRC Building, Room C-312, PO Box 15000, Halifax, Nova Scotia B3H 4R2 Canada; 2 Cardiff University, Cardiff, UK; 3 McGill University, Montreal, Canada; 4 National University of Singapore, Singapore, Singapore; 5 Monash University, Melbourne, Australia; 6 University of Oklahoma Health Sciences Center, Oklahoma City, USA; 7 Erasmus University Rotterdam, Rotterdam, The Netherlands

**Keywords:** Human Genome Organisation, Genomics, Solidarity, The public good, Benefit sharing, Bioinformatics

## Abstract

The HUGO Committee on Ethics, Law and Society (CELS) undertook a Working Group exploration of the key ethical issues arising from genome sequencing in 2013. The *Imagined Futures* paper the group subsequently published proposed points to consider when applying genomic bioinformatics to data repositories used in genomic medicine and research (http://www.hugo-international.org/Resources/Documents/CELS_Article-ImaginedFutures_2014.pdf). Given the ever-increasing power to sequence the human genome rapidly and inexpensively—as well as trends toward “Big Data” and “Open Science”—we take this opportunity to update and refine the key findings of that paper.

## Introduction

In 2013, the Human Genome Organisation’s (HUGO) Committee on Ethics, Law and Society (CELS) undertook a Working Group exploration of the key ethical issues arising from genome sequencing, focusing on bioinformatics: an umbrella term linking biological data with techniques for information storage, access, and analysis to support multiple areas of scientific research and clinical treatment.^1^ The following is a HUGO Statement based on the *Imagined Futures* paper the Working Group published, in which a framework for oversight of genomic repositories was developed. This Statement provides an ethical and philosophically consistent basis to regulate and apply bioinformatics in genomic research and clinical practice. The Working Group developed the following principles:^2^

Social justice: Based on HUGO’s affirmation to “adhere to international norms of human rights” and HUGO’s *leitmotiv* to recognize the “genome as the common heritage of humanity,” social justice is the right of every individual to share in the benefits of scientific progress and its technological applications.^3^ The strongest statement on social justice from HUGO can be found in the *Statement on Benefit Sharing* (2000):

“At present there is a great inequality between the rich and poor nations in the direction and priorities of research and in the distribution and access to the benefits thereof. When there is a vast difference in power between those carrying out the research and the participants, and when there is a possibility of substantial profit, considerations of justice support the desirability of distributing some profits to respond to health care needs.”

Genomic solidarity: HUGO’s first articulation of genomic solidarity also came in its *Statement on Benefit Sharing*, which stated:

“The sharing of genes may call for strong solidarity within certain groups of people. Members of a small group with rare genes who have helped research would be particularly deserving recipients of benefits. … It is in everyone’s best interest that wealthy and powerful nations as well as commercial entities foster health for all humanity.”

This principle naturally follows from social justice because differences between individuals, communities, and populations, revealed by genomic research, should not limit fair opportunities or lead to discrimination. Therefore, genomic research should be a reciprocal exchange between individuals and communities, with researchers, funders, and sponsors, so that all participants (human beings as originators of sequences) share in the benefits of the research through knowledge dissemination and progress, and not just as end-product users, for the reason that may create inequity because of commercial interests and differential access. Solidarity, however, is reciprocal with the “right to the protection of the moral and material interests resulting from any scientific, literary or artistic production of which [they are] the author.”^4^ Practicing solidarity thus also involves: upholding quality research as correlative to it being ethical; establishing scientists’ accountability to funders, participants, and publics; and societal support for bona fide scientists in their contributions to the public good.^5^

“For the public good”:^6^ All social endeavors—including genomic research—should benefit individuals as members of the community of rights.^7^ Here, equity involves working to reduce health inequalities among different populations by promoting equal access to the benefits of scientific progress.^8^ Equity should be a prerequisite for translating genomic knowledge into socially and morally valued public goods. In focusing on the public good, we believe that future regulatory models should be aimed at generating public social benefit and not merely private commercial gains.

### The *Imagined Futures* paper

*Imagined Futures* focused on three “futures” (see the “Appendix” section):

1. Clinical indications for whole genome sequencing,
2. Cancer genomics in diagnosis and therapeutic guidance, and
3. Microbial genomics and metagenomics.

For each of these topics, the Working Group systematically examined how the translation of genome science could occur in a way that positively “captures” the public good: conditions for knowledge production, and its access and use for morally worthy purposes. We analyzed which conditions were necessary for the benefits of these imagined futures to materialize and which potential barriers existed toward those goals.

In all three futures, bioinformatics will have a central role in creating opportunities for genomics to benefit society. Such outcomes will depend on appropriate regulation and clinical governance of some complex tasks, and significantly, the creation and management of *data repositories*. While the “imaginaries” are potentially prophetic, the reality of the technical aspects of genome sequencing is significant. In this respect, the *Imagined Futures* paper focused on how bioinformatics, as a technical consideration of data collection and storage in large scale repositories, requires consideration of ethical, legal, and social oversight.

The genomic “revolution” relied on developing powerful sequencing technologies and the concomitant advances of computational capabilities, and it is therefore important to note the technical limitations and challenges of bioinformatics:

- Clinical sequences must be accurate and with very low error rates.
- Even comparatively low-cost whole genome sequencing could remain too expensive for common use in health care for countries with limited resources.
- Specific standards are needed to calibrate sensitivities and specificities across heterogeneous technical platforms.

With ever-increasing power to sequence the human genome rapidly and inexpensively, come challenges associated with “Big Data” and “Open Science,” and in these respects, ethical frameworks for bioinformatics will be fundamental. While Big Data increases the scale and ease of analyzing troves of data to reveal patterns, Open Science changes what is knowable, accessible, and useable in terms of making scientific research more socially just. As both raise questions about the ethical conditions for data collection and usage, the public good is likely to play a key role in creating new paradigms for safeguards and oversight of data collection and storage.

#### The Statement

##### Imagining genomic data repositories

When patients, clinicians, and researchers entrust genomic data to a data repository, they generally want assurances that:

- The information will be available when needed.
- The information is accurate, correct, and validated.
- The information will be shared with others and used in an appropriate manner.^9^

We imagine an approach in which a person’s whole genome is sequenced at one time, and then stored in a *trusted* genomic repository. Different designated pathways will be “gated” to protect the interests in “clinical,” “research,” and “public health.” These three pathways will each require the clarification of specific and shared ethical and legal conditions. All three will require similar bioinformatics considerations regarding data intake, data use, long-term storage, translation, and resolving threats to the repository (e.g., disasters, human errors, hacking, and computational attacks, “bit rot,”^10^ and format obsolescence). Therefore, the *Imagined Futures* model will generally require that:

- Only complete genome sequences with appropriate accuracy metrics will be deposited in the data repository.
- This repository has reasonable and up-to-date privacy and security safeguards.
- The data are stored in a stable format and use ethically and legally sustainable process of access.
- Indications for new sequencing are clear, with inevitable technical innovations.
- Depending on the analysis schedule, sequences from specified regions of the genome will be accessed, analyzed, and interpreted by professional, skilled genomic analysts.

##### Recommendations

Recognizing that:

i. Genetic determinism does not reflect the range and complexity of factors (including genes) that influence human health and development.
ii. Social justice can promote the public good by challenging the vagaries of the for-profit sector in genomic research (i.e., recognizing and admonishing conflicts of interest).
iii. The complexity of the data gives rise to technical challenges, as well as problems of its interpretation both as clinical and biological information.
iv. Practical solutions to these challenges are needed in order to maximize potential public goods. These may include technical, professional, educational, and regulatory approaches.
v. Potential benefits should not be confined to specific regions of the world, such as those rich in resources.
vi. The protection of individual and community rights should be balanced with the broader interests of society (including the right to benefit from scientific advancement).^11^
vii. Regulation can improve public trust in new technology by letting people know that its developments and uses are being monitored and that controls and sanctions are in place to prevent misuse.

Subject to these *Imagined Futures* becoming real, the HUGO Committee on Ethics, Law and Society *recommends* that:

1. Human genome sequences should be stored in a safe and long-term professional environment. It should be retrievable, either in whole or in part, based on the appropriate scientific and ethical conditions for research, clinical practice, and public health:
  i. The trusted genomic repository (TGR) as a whole must be run by a trustworthy entity, i.e., a custodian who diligently applies appropriate policies and reacts appropriately when the policies are breached;
  ii. As part of its data quality control procedures, the TGR should only accept data from sources it considers valid and trustworthy;
  iii. The TGR must have documented, well-understood policies stating who is allowed to do what to which data, for which purpose, and when;
  iv. The TGR must have policies regarding how long data will be retained and when and how they should be purged;
  v. The repository must follow standard best practices for mitigating known threats and evolve to address new ones;
  vi. Contributors and patients should know how their genome sequences may be accessed and for what reasons, and that technical limits to anonymity mean that some risks will always remain;
  vii. There should be a new profession for *computationally enabled genomic analysts* or “genomic bioinformatician.” Accreditation of that role will set the standards for interpretive genomic medicine and research.
2. Consideration should be given to the establishment of a forum for global consensus building related to TGRs as they develop links to one another—perhaps under the auspices of HUGO.
3. HUGO should continue to promote international norms of human rights as the basis for public health, recognizing that specific regulatory measures should be kept up to date, and should not obstruct science or become isolated from the public’s views on the technologies or applications.

In research applications,

4. Jurisdictions should consider the merits of establishing consolidated databases for full genome sequences of human and non-human animals, as well as relevant plants, bacteria, and viruses, as a resource for public health.^12^
5. Regulations should be compatible with best practices and harmonized with international standards to ensure that there is no weak link in the regulatory chain.

In clinical applications,

6. Genomic sequencing technologies should be translated into the medical practice in ways that reflect the complementarity of personalized medicine with the collective benefits of public health.
7. Terminology and indications for redoing a clinical sequence are not settled. “Re-sequencing” is starting a new biochemical assay with a new sample or DNA from the original sample. “Re-interpretation” is taking the first sequence but putting it through new analytic (computer) algorithms or rewriting the clinical report in view of new literature on the variants seen. Issues of all new sequencing chemistries should be raised as well as sequencing other samples, such as RNA sequencing from actual diseased tissue (cancer or other).
8. Genome sequencing standards should be established by professional or national metrology groups to ensure:
  i. The high quality of each individual/’s genome sequence;
  ii. The quality of clinical interpretation for these data;
  iii. That health care professionals have well-defined obligations and responsibilities as legal owners or stewards of the genomic data, circumstances in which the data is provided and collected, and in respect to the conditions for inclusion in the data and the patients it pertains to.

We note that the research, clinical, and public health domains are not mutually distinct.

## Concluding remarks

The original *Imagined Futures* noted that the context of existing international systems considerably complicates any singular regulatory model and its effectiveness. Complexity stems from economic, ethical, legal, and social permutations; terminological distinctions and boundaries; and conditions appropriate for various different forms of specimens, information, and code or formula, that may be stored alongside genomic sequences. Socio-economic and cultural differences are amplified as regulators transition from local contexts, through to regions of cohabiting communities, and finally internationally. This Statement, therefore, is intentionally broad to recognize challenging regulatory environments and to focus on areas in which there is inadequate oversight.^13^ Advances in science and technology have become key drivers in international relations; thus, this Statement may form the basis of these relations and cooperation with respect to ethical bioinformatics in genomics. The Statement does not preclude a role for other international bodies involved in standard setting in the genomics area; these can be effectively drawn on to provide further and strengthened cooperation. There are also a range of international research organizations (e.g., International Stem Cell Consortium) that have developed guidelines in specific fields that, within a particular fraternity of professionals, provide harmonized standards for research and clinical practice. HUGO encourages national and international regulatory bodies to continue to work together, such as the recent joint initiatives of the Human Variome Project and HUGO. Although individual responses to this Statement will depend on individual countries, organizations, and initiatives, HUGO believes that fundamental values must be the same in the achievement of our imagined future for the public good.

## Appendix

### The *Imagined Futures*

#### Imagined future 1: clinical indications for whole genome sequencing

Complete sequence information of an individual can provide a great deal of clinically useful information concerning disease risk, treatment response, and risk for adverse drug effects. These genetic data must be reinterpreted, even re-sequenced, as new clinical and technical information arises. Assuming that a sequence will be definitively assayed at least once in life (e.g., at birth), this scenario imagines when and how health care professionals will query an individual’s stored sequence.

#### Imagined future 2: cancer genomics in diagnosis and treatment

Biomarkers and therapeutic markers already play a major role in clinical oncology. Our scenario further imagines that genomics will become routinely employed in the clinical management of cancers and probably other disorders.

#### Imagined future 3: microbial genomics and metagenomics

Microbial genomics is the sequencing, analysis, interpretation, and application of genomic data from individual bacterial, viral, and other pathogenic agents. Metagenomics is the study of sequence information from microbial communities, including those that colonize human tissues and organs such as the gut, lungs, skin, and nasopharynx. In the third scenario, sequence-based diagnostics incorporating this information tailors therapeutics for optimizing individual patient outcomes, understanding local epidemiology, and improving community health.

## Footnotes

1 Capps, B., Chadwick, R. (Chair), Chalmers, D., Clarke, A., Clayton, E.W., Liu, E. (Co-chair), Lysaght, T., Mulvihill, J. and Winslett, M. 2013. Imagined Futures: capturing the benefits of genome sequencing for society. Report prepared by the working group of the HUGO Committee on Ethics, Law and Society. December. Accessible on the website of the Human Genome Organisation: *http://www.hugo-international.org/HUGO-CELS*

2 These are emergent precepts emanating from first principals originally cited in the Statement on the Principled Conduct of Genetics Research (1995), and periodically reviewed in proceeding HUGO Statements: (1) recognition that the human genome is part of the common heritage of humanity; (2) adherence to international norms of human rights; (3) respect for the values, traditions, culture, and integrity of participants; and (4) acceptance and upholding of human dignity and freedom. The current principles are contemporary with extant, and “next generation” ethical norms in today’s genomic practices. All HUGO Statements are accessible on the website of the Human Genome Organisation: *http://www.hugo-international.org/HUGO-CELS*

3 Article 27 of the Universal Declaration of Human Rights (UDHR, 1948) and Article 15 of the International Covenant on Economic, Social and Cultural Rights (ICESCR, 1966).

4 Ibid.

5 Capps, B. 2013. Defining variables of access to UK Biobank: the public interest and the public good. *Law, Innovation and Technology* 5: 113-139.

6 To be distinguished from the common or greatest good, which likely indicate a utilitarian or economic assessment of collective benefits.

7 As members of the community of rights “…we should treat ourselves as having both rights and responsibilities with regard to the protection, preservation, and promotion of the generic context for agency (that is to say, the agency commons). We can argue about the details of this context (or commons), but it will include elements pertaining to our well-being (clean air and water, food, environmental integrity, and the like) and our freedom (security, an absence of fear and intimidation, and so on). Not to harm one another directly is fine but the setting for flourishing agency requires more than this minimal interactive restraint.” Brownsword, R. 2009. Friends, Romans, and Countrymen: is there a universal right to identity? *Law Innovation and Technology* 1: 223-249. p. 229.

8 In *Imagined Futures*, as well as two subsequent background papers, the concept of the public good—as defined here—took a different meaning from that previously used by HUGO; see: Capps, B., Chadwick, R., Joly, Y., Mulvihill, J., Lysaght, L. and Zwart, H. 2017. Falling giants and the rise of gene editing: ethics, private interests and the public good. *Human Genomics* 11:20; DOI 10.1186/s40246-017-0116-4; Mulvihill, J., Capps, B., Joly, J. Lysaght, T., Zwart, H. and Chadwick, R. The International Human Genome Organisation (HUGO) Committee of Ethics, Law, and Society (CELS). 2017. Ethical Issues of CRISPR Technology and Gene Editing. *British Medical Bulletin* 122: 109-122. Previous statements emanating from HUGO have affirmed both the human genome and human genomic databases as “global public goods” as “those whose scope extends worldwide, are enjoyable by all with no groups excluded, and, when consumed by one individual are not depleted for others”; Knoppers, B., Thorogood, A. and Chadwick, R. 2013. The Human Genome Organisation: towards new-generation ethics. *Genomic Medicine* 5: 38; HUGO Ethics Committee: Statement on Human Genomic Databases 2002.

9 See: Ekong, R., Vihinen, M. and Parker, M. Human Variome Project. 2018. Ethical Data Management: Checklist for Gene/Disease Specific Database Curators. Human Variome Project Working Group WG08.

10 Cerf, V. 2011. Avoiding “Bit Rot”: Long-term preservation of digital information. *Proceedings of the IEEE* 99: 915-916; Dickmann, F., Grütz, R., and Rienhoff, O. 2012. A "meta"-perspective on "bit rot" of biomedical research data. *Studies in Health Technology and Informatics* 180: 260-264.

11 Knoppers, B., Thorogood, A. and Chadwick, R. 2013. The Human Genome Organisation: towards new-generation ethics. *Genomic Medicine* 5: 38.

12 Capps, B. and Lederman, Z. 2015. One Health and paradigms of public biobanking. *Journal of Medical Ethics* 41: 258-262.

13 “Regulation seldom starts at the international level: nation states have to develop rules for their own societies before they can even begin to think about creating an international regulatory system... but there is absolutely no reason to rule out the possibility that it will emerge at this early stage in the game”; Fukuyama, F. 2002. *Our Posthuman Future: Consequences of the Biotechnology Revolution*. London: Profile Books. p. 191.

